# Tregs at Diagnosis as a Potential Biomarker for Predicting High‐Risk Functionality in Newly Diagnosed Multiple Myeloma

**DOI:** 10.1002/cam4.70980

**Published:** 2025-06-03

**Authors:** Qiaolin Zhou, Fang Xu, Jingjing Wen, Jing Yue, Ya Zhang, Lijun Du, Kun Kou, Jing Su, Yiping Liu, Xiaogong Liang

**Affiliations:** ^1^ Hematology Department, Mianyang Central Hospital, School of Medicine University of Electronic Science and Technology of China Mianyang China

**Keywords:** biomarker, early relapse, functional high risk, multiple myeloma, newly diagnosed, prognosis, Treg

## Abstract

**Objective:**

This study investigated the role of regulatory T cells (Tregs) in newly diagnosed multiple myeloma (NDMM) patients, particularly in relation to early relapse and prognosis.

**Methods:**

The analysis included clinical data from 70 NDMM patients, with Tregs measured at diagnosis. Early relapse was defined as relapse within 18 months (ER18), and posttransplant survival extending beyond 12 months. Functional high risk (FHR) was evaluated based on this criterion.

**Results:**

For the overall cohort, the median progression‐free survival (PFS) and overall survival (OS) were not reached, but in the ER18 cohort, median OS was 24.8 months and median PFS was 10.8 months. Key factors linked to early relapse included elevated serum creatinine levels (> 156 μmol/L), presence of extramedullary disease, and lower percentage of Tregs at diagnosis. Multivariate analysis revealed that extramedullary disease and lower percentage of Tregs were significant predictors of early relapse. Factors such as age, elevated creatinine, extramedullary disease, and lower percentage of Tregs were associated with poorer PFS. Further analysis confirmed that extramedullary lesions, elevated creatinine, and lower percentage of Tregs significantly influenced PFS.

**Conclusion:**

Overall, Tregs at diagnosis were found to be important for predicting early relapse and progression‐free survival, highlighting their potential as a biomarker for functional high risk in multiple myeloma.

## Graphical abstract

Tregs levels at diagnosis were assessed to predict early relapse (ER18) and functional high risk in multiple myeloma. Our findings suggest that Tregs at diagnosis could serve as a potential biomarker for early relapse and long‐term prognosis in newly diagnosed patients.

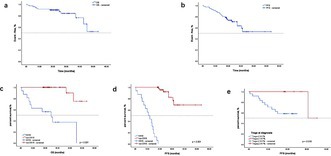

**Abbreviations**
ASCT: autologous hematopoietic stem cell transplantation
CR: complete response
ER: early relapse
ER18: early relapse within 18 months
FHR: functional high risk
IMIDs: immunomodulatory drugs
IMWG: International Myeloma Working Group
LTS‐MM: long‐term survival MM
MM: multiple myeloma
MRD: minimal residual disease
NDMM: newly diagnosed multiple myeloma
OS: overall survival
PBMCs: peripheral blood mononuclear cells
PFS: progression‐free survival
PIs: proteasome inhibitors
PR: partial response
Tregs: regulatory T cells
VCD: bortezomib plus cyclophosphamide and dexamethasone
VGPR: very good partial response
VRD: bortezomib plus lenalidomide and dexamethasone

## 1 Introduction

Multiple myeloma (MM) is one of the most frequent hematological malignancies with great heterogeneity [1]. Despite significant improvements in the prognosis of MM through the use of proteasome inhibitors, immunomodulators, and CD38 monoclonal antibody, the disease remains incurable and requires ongoing treatment [2, 3]. Almost all newly diagnosed multiple myeloma (NDMM) patients eventually relapse due to the development of drug resistance and persistent residual disease. Based on the classic and cytogenetic risk stratification, 11.6% of patients classified as non‐high‐risk by traditional stratification methods may still experience early progression [4]. Therefore, identifying patients with functional high risk (FHR) early has brought increased attention. FHR MM represents a subset of patients with an aggressive disease course characterized by early relapse (ER), even when treated with novel agents including proteasome inhibitors (PIs) and/or immunomodulatory drugs (IMiDs), autologous stem cell transplantation (ASCT) and CD38 antibody [4, 5, 6]. The complexity of treating MM with FHR arises from the heterogeneity of the disease and its resistance to treatment. However, to our knowledge, the definition of FHR is mainly based on the clinical outcome of early relapse. It has become a key problem how to recognize NDMM patients with FHR, except for the cytogenetics and other known biomarkers, before acquiring the survival outcome.

Abnormalities of the tumor microenvironment are involved in the progression of MM. The immune cells at the site of the microenvironment are vital determinants of disease evolution. One of the major immunosuppressive mechanisms in tumor progression is the expansion of regulatory immune cells, particularly Tregs [7]. Tregs are crucial immune suppressor cells, playing a critical role in various immune disorders and tumors. As reported, in MM, Tregs play various roles, including participating in the onset and progression of the disease, maintaining disease homeostasis, and correlating with prognosis [8, 9, 10]. They also play an important role in the maintenance of self‐tolerance and the control of immune homeostasis; however, they can be coopted by cancer cells to aid in immune suppression and evasion. Tregs can inhibit tumor‐specific CD8^+^ and CD4^+^ T cell effector functions through cell–cell contact and/or the production of anti‐inflammatory cytokines [7]. The immune status is closely associated with the pathogenesis and prognosis of MM; Tregs, as essential immune cells, play a significant role. The literature reports that Tregs may be associated with ER [11]. Therefore, we hypothesize that they are related to FHR and may help in the early identification of FHR and guide subsequent treatment. This study investigated that Tregs at diagnosis have a potential role in prognostic assessment and in identifying FHR patients in NDMM.

## 2 Methods

### 2.1 Patients

A total of 70 NDMM patients were included in this study; their clinical information was collected from medical records in our hospital from 2016 to 2023. The diagnosis of MM was established following the criteria of the International Myeloma Working Group (IMWG) [12]. Sample collection and review of clinical records were conducted with the participants' informed consent, following the principles outlined in the Declaration of Helsinki.

### 2.2 Study Treatment

Patients were assigned to two treatment groups: VCD (bortezomib, cyclophosphamide, dexamethasone) and VRD (bortezomib, lenalidomide, dexamethasone). VCD involved 35‐day cycles of subcutaneous bortezomib (1.3 mg/m^2^ on Days 1, 8, 15, 22), intravenous cyclophosphamide (300 mg/m^2^ on Days 1, 8, 15), and intravenous dexamethasone (20 mg on Days 1, 2, 8, 9, 15, 16, 22, 23). VRD followed a similar bortezomib and dexamethasone schedule, with oral lenalidomide (10 or 25 mg daily for 21 days). Treatment protocols were agreed upon by clinicians and patients.

### 2.3 Flow Cytometric Analysis

Peripheral blood samples anticoagulated with EDTA were collected, and Tregs (CD3^+^CD4^+^CD25^+^FOXP3^+^) were analyzed in this study. Flow cytometry was utilized to determine the frequencies of Tregs. For Treg analysis, the thoroughly mixed and processed sample was carefully transferred based on the white blood cell count results. Red blood cells were lysed using a lysis buffer (Beyotime, catalog C3702), ensuring complete removal from the whole blood sample. The remaining cells were then stained for surface markers, including CD45 PerCP (catalog 045‐104‐3/clone 4A, Biotech), CD3 FITC (catalog 340542/clone SK7, BD), CD4‐PEcy7 (catalog 348799/clone SK3, BD), and CD25 APC (catalog 567316/clone bc96, BD). For intracellular transcription factor FoxP3 staining, the FoxP3/Transcription Factor Staining Buffer Set Kit (eBiosciences, catalog 00‐5523‐00) was used according to the manufacturer's instructions. Cells were fixed, permeabilized, and incubated with FOXP3 PE (catalog 12‐4776‐42/clone PCH101, Thermo Fisher) for 60 min at room temperature in the dark.

The percentages of Tregs were calculated relative to the total CD3^+^CD4^+^ T cell population. Samples were analyzed using a BD FACSCanto flow cytometer (BD Biosciences), and data were processed using DIVA software. The detection of Tregs was conducted by Sichuan West China Kang Shengda Medical Testing Co. Ltd.

### 2.4 Assessments

Response assessments were performed according to the IMWG response criteria [12]. Early relapse was defined as occurring within 18 months after initial treatment or within 12 months after ASCT (ER 18) [4]. We also refer to this entity as functional high‐risk MM (FHRMM) [11], while relapse occurring beyond 18 months, with overall survival exceeding 18 months and posttransplant survival extending beyond 12 months, was classified as late relapse (non‐ER18). Treg detection was conducted at the time of diagnosis, thereby excluding any potential influence of ASCT on Tregs. The efficacy assessments were all conducted before ASCT; all assessments were conducted after 3–4 treatment cycles. The primary objective of this study was to evaluate early relapse. Secondary objectives included progression‐free survival (PFS) and the best response posttreatment, which encompassed complete response (CR), very good partial response (VGPR), and partial response (PR). Additional outcomes assessed were the disease progression rate and the predictive factors for ER18 and PFS.

### 2.5 Follow‐Up

All patients were monitored for disease progression or mortality. The most recent follow‐up for surviving patients was conducted on May 24, 2024. Cases that were lost to follow‐up were excluded. The duration from the initial diagnosis to disease progression or mortality unrelated to progression was designated as PFS. The timeframe from the initial diagnosis to the last follow‐up or mortality was defined as overall survival (OS).

### 2.6 Statistical Methods

All experimental data in this study were analyzed using SPSS 23.0 and GraphPad Prism 8.0. Patient‐defined demographics, disease characteristics, and treatment details were analyzed using descriptive statistics. Quantitative data were assessed for normality using the Shapiro normality test. Normally distributed data were presented as χ ± S; non‐normally distributed data were presented as median. Categorical variables and continuous variables were analyzed using Fisher's exact test and the Wilcoxon run‐sum test, respectively. The log‐rank test and Kaplan–Meier method were used to estimate the time‐to‐event endpoints and between‐group comparisons for PFS. Cox regression was used for the univariate survival analysis and multivariate survival analysis. Variables with a *p* value < 0.05 in the univariate analysis were included in the multivariate models. All *p* values were two‐sided; *p* < 0.05 was considered to indicate a statistically significant result.

## 3 Results

### 3.1 Patient Background

Seventy NDMM patients were included in this study; baseline demographic and disease characteristics are summarized in Table 1. The median age was 63 (range: 45–87) years. Among the 70 patients, cytogenetic and FISH analyses showed that 36.0% of the patients were at high cytogenetic risk, including 20.8% of patients with del 17p, 2.0% with t(14;16) and 26.5% with t(4;14), and no patients were concurrent with t(14;20). The percentage of patients with extramedullary lesions was 10.0%. This cohort consisted of 16 patients (22.9%) who experienced ER18. Compared to the group late relapse, patients in the ER18 group had a higher proportion of older age (*p* = 0.027), elevated serum creatinine (*p* = 0.005), lower ASCT rate (*p* = 0.037), and lower percentage of Tregs at diagnosis (*p* = 0.011), as shown in Table 2.

**TABLE 1:** Baseline clinical characteristics of the patients.

Characteristics	Patients (*N* = 70)
Median age (range)	63 (45, 87)
Age group, *n* (%)	
60 years	28 (40.0%)
≥ 60 years	42 (60.0%)
Male, *n* (%)	43 (61.4%)
ECOG performance status, *n* (%)	
≥ 2	15 (21.7%)
ISS stage at diagnosis, *n* (%)	
I	18 (25.7%)
II	26 (38.2%)
III	24 (35.3%)
Hemoglobin < 100 g/L	34 (48.6%)
Platelet < 100 × 10^9^/L	11 (15.7%)
Median LDH, U/L (range)	185.00 (88.00, 502.00)
Median serum creatinine, μmol/L (range)	83.8 (35.60, 1114.30)
Cytogenetic abnormalities, *n* (%)	
Del17p	10 (20.8%)
t(14;16)	1 (2.0%)
t(4;14)	13 (26.5%)
+1q21	29 (58.0%)
Extramedullary disease, *n* (%)	7 (10.0%)

**TABLE 2:** Comparison of clinical features between non‐ER18 and ER 18.

Clinical features of patients	Non‐ER18 (*n* = 54)	ER18 (*n* = 16)	*p*
Male, *n* (%)	34 (62.96)	9 (56.25)	0.628
Mean age	61.74 ± 10.66	67.44 ± 7.98	0.027
ECOG performance status, *n* (%)			
≥ 2	14 (26.42)	1 (6.25)	0.171
Median β_2_‐microglobulin	3.7 (2.54, 5.65)	4.5 (3.33, 5.8)	0.332
Median LDH	184 (152, 232)	191 (137.5, 211.75)	0.405
High‐risk cytogenetic abnormalities,^a^ %	13 (37.14)	5 (33.33)	0.797
t(14; 20), *n* (%)	NA	NA	NA
t(4; 14), *n* (%)	9 (25.71)	4 (28.57)	1.000
t(14; 16), *n* (%)	1 (2.86)	0 (0)	1.000
del 17p, *n* (%)	6 (18.18)	4 (26.67)	0.774
1q21, *n* (%)	21 (60.00)	8 (53.33)	0.662
ISS			0.096
I	17 (32.69)	1 (6.25)	
II	19 (36.54)	7 (43.75)	
III	16 (30.77)	8 (50.00)	
Serum creatinine ≥ 156 μmol/L, *n* (%)	7 (12.96)	5 (50.00)	0.005
Extramedullary disease, *n* (%)	3 (5.56)	4 (25.00)	0.071
Initial therapy			0.098
PI‐based	18 (33.33)	9 (56.25)	
PI and IMIDs	36 (66.67)	7 (43.75)	
Maintenance treatment			1.000
PI‐based	13 (24.07)	1 (16.67)	
IMIDs‐based	12 (22.22)	1 (16.67)	
PI and IMIDs	29 (53.75)	4 (66.67)	
ASCT	22 (40.74)	2 (12.50)	0.037
Response to induction			
≥ CR	11 (20.37)	4 (30.77)	0.662
≥ VGPR	31 (57.41)	9 (69.23)	0.435
≥ PR	52 (96.30)	11 (84.62)	0.167
Median Treg%^b^ at diagnosis	2.6 (2.01, 3.91)	1.46 (1.32, 2.41)	0.011

Abbreviation: ASCT, autologous hematopoietic stem cell transplantation.

^a^ High‐risk cytogenetic abnormality was detected by fluorescence in situ hybridization (FISH) or metaphase cytogenetics, including del 17, t(4;14) and t(14;16).

^b^ Treg% were defined as CD4^+^CD25^+^FoxP3^+^/CD4^+^.

### 3.2 Response Rate and Survival Outcome in Entire Cohort

**FIGURE 1:**
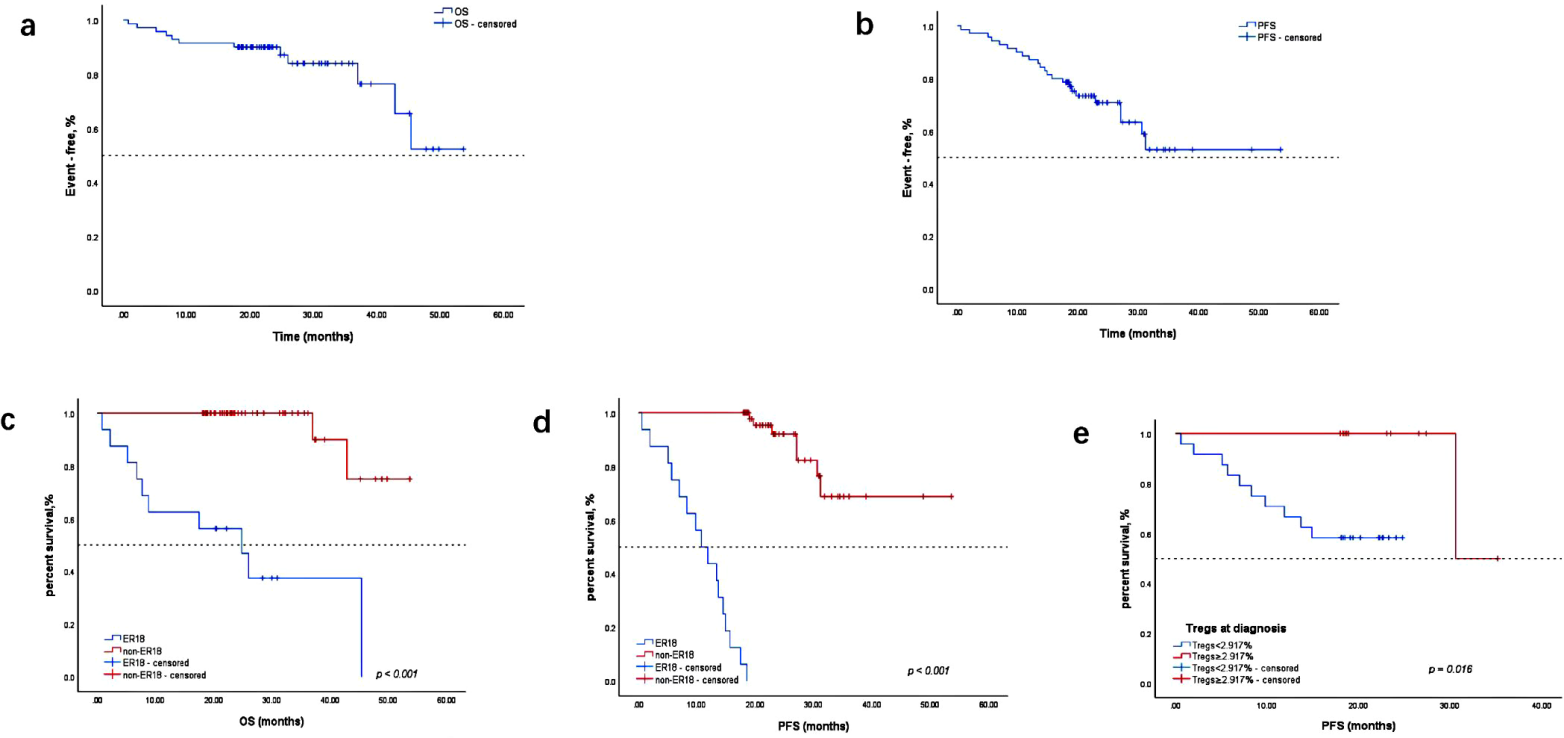
Kaplan–Meier survival curves illustrating survival outcomes in the study cohort. (a) Overall survival (OS) for all patients. (b) Progression‐free survival (PFS) for all patients. (c) OS stratified by ER18‐based risk classification, showing significant prognostic separation between risk groups. (d) PFS stratified by ER18‐based risk classification. (e) PFS stratified by regulatory T cell (Treg) levels at diagnosis, demonstrating the prognostic value of baseline Treg status. Survival differences between groups were assessed using the log‐rank test, and statistical significance was defined as *p* < 0.05.

The best outcome assessment was that 22.4% achieved CR, 59.7% achieved VGPR, and 94.0% achieved PR. PFS and OS for the entire cohort are shown in Figure 1a,b. Among the 70 patients, the median PFS and OS were not reached. At the data cutoff, a total of 23 (32.9%) patients experienced disease progression, and 16 (22.9%) patients experienced ER18.

### 3.3 Prognostic Factors in Multiple Myeloma

In the ER18 group, the median OS was 24.8 months, and the median PFS was 10.8 months. The death rate was significantly higher in the ER18 group compared to the non‐ER18 group (Log‐rank *χ* ^2^ = 35.02, *p* < 0.001), as shown in Figure 1c. Conversely, the ER18 group showed a higher PFS rate (Log‐rank *χ* ^2^ = 109.71, *p* < 0.001), as shown in Figure 1d.

**FIGURE 2:**
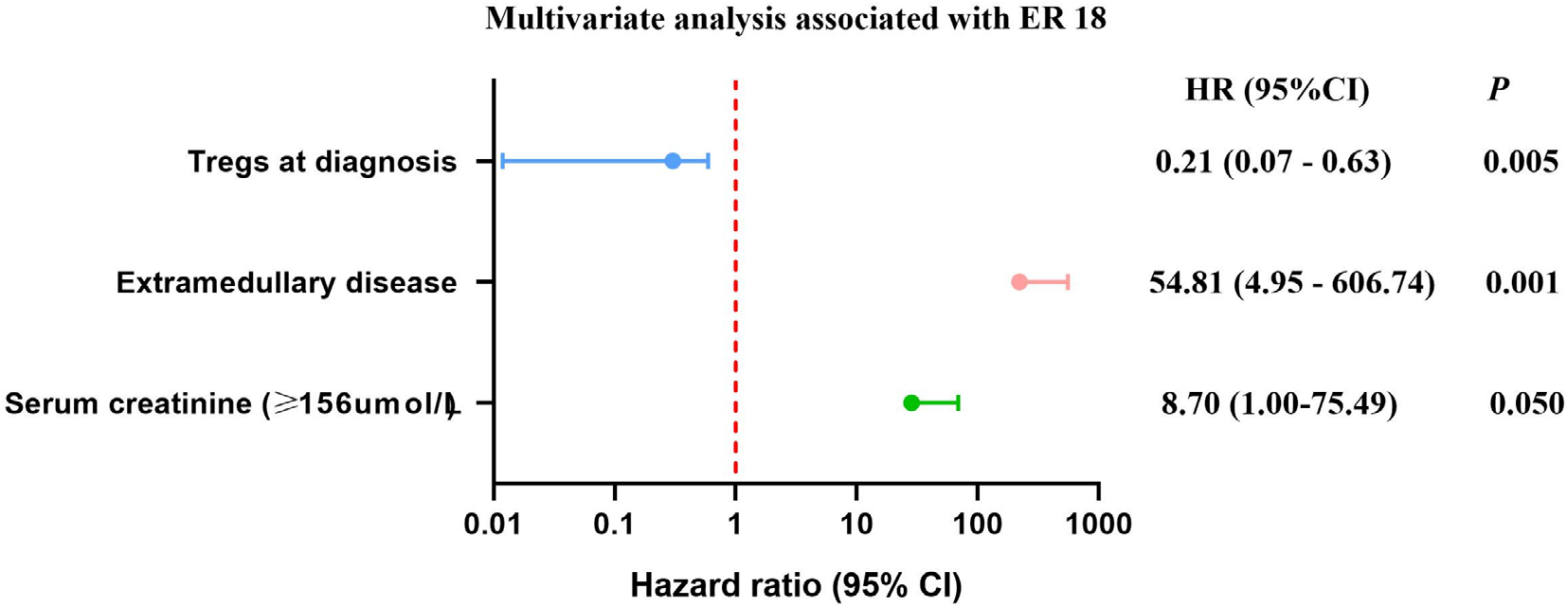
Multivariate analysis associated with ER18.

Baseline clinical characteristics affecting early relapse risk were analyzed, with Table 3 presenting significant parameters identified through univariate logistic regression. Key risk factors included elevated serum creatinine (> 156 μmol/L), extramedullary disease, and lower percentage of Tregs at diagnosis. These variables were further assessed in multivariate analysis, revealing that extramedullary lesions (*p* = 0.001, HR 54.81, 95% CI 4.95–606.74)constitute an independent risk factor for ER18, whereas a higher percentage of Tregs (*p* = 0.005, HR, 95% CI 1.49–432.97) serves as an independent protective factor against ER18, as shown in Figure 2.

**TABLE 3:** Prognostic factors associated with ER18 identified through univariate analysis.

	Odds ratio	95% confidence interval	*p*
Sex (male)	0.77	0.29–2.07	0.602
Age	1.05	1.00–1.10	0.055
ISS (II + III)	6.19	0.82–46.86	0.078
β2‐microglobulin	1.03	0.94–1.12	0.586
Hb < 100 g/L	1.94	0.70–5.34	0.200
PLT < 100 × 10^9^/L	1.28	0.36–4.50	0.699
Serum creatinine > 156 μmol/L	4.42	1.66–11.80	0.003
Calcium	0.22	0.02–2.07	0.184
LDH	1.00	0.99–1.01	0.866
Extramedullary disease	4.85	1.55–15.17	0.007
High risk	0.84	0.29–2.46	0.749
Cytogenetics			
t(14; 20)	NA	NA	NA
t(4; 14)	1.11	0.35–3.55	0.858
t(14; 16)	0.00	0.00—Inf	0.998
del 17p	1.4	0.45–4.40	0.578
1q21	0.80	0.29–2.22	0.672
Treg%^a^ at diagnosis	0.32	0.12–0.88	0.028
Initial therapy (within IMIDs regimen)	0.44	0.16–1.18	0.104
Maintenance treatment			1.000
PI‐based	Ref.		
IMIDs‐based	1.14	0.07–18.31	0.924
PI and IMIDs	1.78	0.20–15.96	0.605
ASCT	0.23	0.05–1.00	0.050
Response to induction			
≥ CR	1.45	0.45–4.70	0.539
≥ VGPR	1.44	0.44–4.69	0.541
≥ PR	0.24	0.05–1.07	0.062

^**a**^ Treg% was defined as CD4^+^CD25^+^FoxP3^+^/CD4^+^.

**FIGURE 3:**
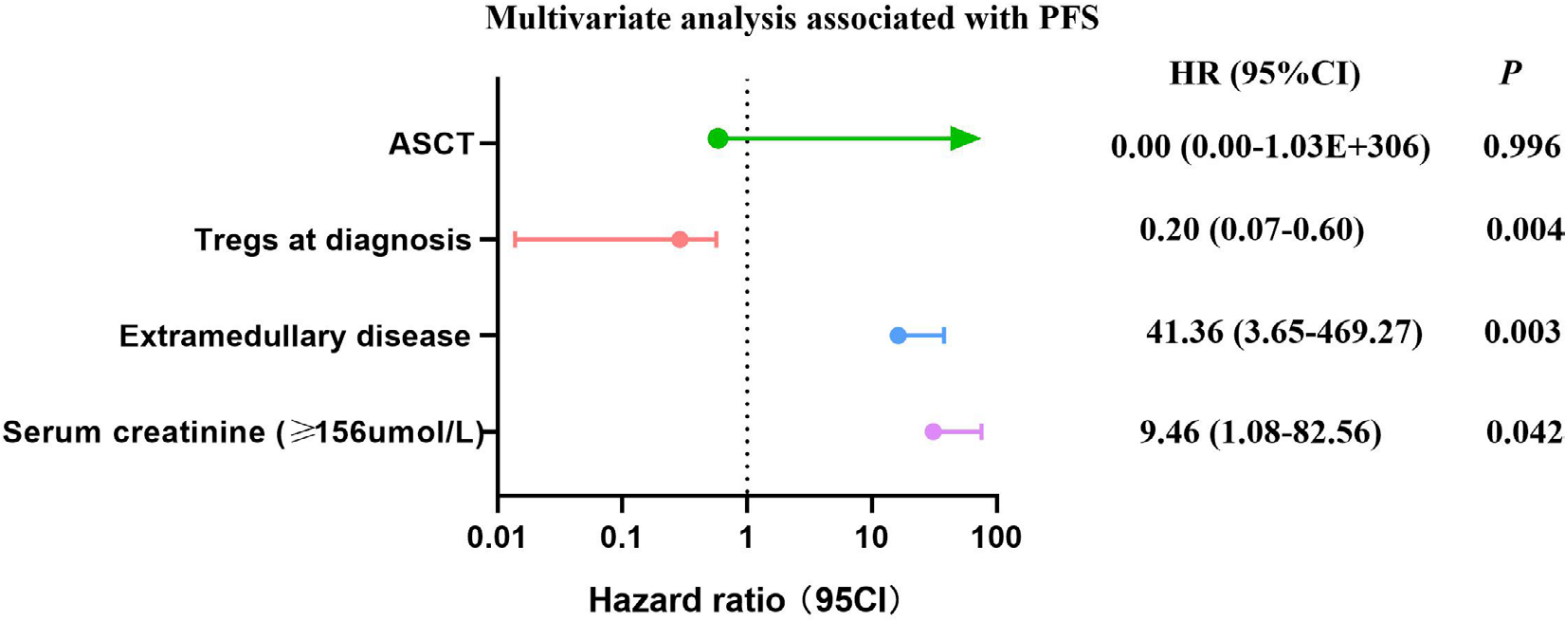
Multivariate analysis associated with PFS.

The overall cohort's median OS and PFS were not reached. Table 4 details clinical parameters significantly associated with survival, analyzed through univariate and multivariate Cox regression. Significant factors affecting PFS included age, elevated creatinine, extramedullary disease, Tregs at diagnosis, and ASCT. Cox analysis confirmed that extramedullary lesions (*p* = 0.003, HR 41.36, 95% CI 3.65–469.27), elevated creatinine (*p* = 0.042, HR 9.46, 95% CI 1.08–82.56), and lower percentage of Tregs (*p* = 0.0004, HR 0.20, 95% CI 0.07–0.60) were significantly linked to adverse prognosis, as shown in Figure 3.

**TABLE 4:** Prognostic factors associated with PFS identified through univariate analysis.

	Hazard ratio	95% confidence interval	*p*
Sex (male)	0.71	0.31–1.63	0.422
Age	1.06	1.02–1.11	0.004
ISS (II + III)	1.76	0.65–4.77	0.268
β2‐microglobulin	1.01	0.92–1.10	0.888
Hb < 100 g/L	1.39	0.61–3.17	0.429
PLT < 100 × 10^9^/L	1.27	0.43–3.75	0.665
Serum creatinine > 156 μmol/L	3.244	1.32–7.94	0.010
Calcium	0.20	0.03–1.36	0.100
LDH	1.00	1.00–1.01	0.689
Extramedullary disease	3.13	1.05–9.32	0.040
High risk	0.76	0.26–2.20	0.616
Cytogenetics			
t(14; 20)	NA	NA	NA
t(4; 14)	1.03	0.33–3.24	0.959
t(14; 16)	0.00	0.00—Inf	0.998
del 17p	1.27	0.41–3.95	0.675
1q21	0.70	0.26–1.87	0.482
Treg%^a^ at diagnosis	0.47	0.22–1.00	0.049
Initial therapy (within IMIDs regimen)	0.55	0.24–1.25	0.154
Maintenance treatment			
PI‐based	Ref.		
IMIDs‐based	0.56	0.14–2.24	0.409
PI and IMIDs	0.31	0.09–1.10	0.069
ASCT	0.14	0.03–0.60	0.008
Response to induction			
≥ CR	1.36	0.52–3.56	0.532
≥ VGPR	1.16	0.46–2.92	0.747
≥ PR	0.30	0.07–1.34	0.116

^**a**^ Treg% was defined as CD4^+^CD25^+^FoxP3^+^/CD4^+^.

### 3.4 Tregs Are Associated With Disease Status and Prognosis in NDMM

The median percentage of Tregs at diagnosis in the ER18 group was 1.46 (1.32, 2.41), significantly lower than 2.6 (2.01, 3.91) in the non‐ER18 group (*p* = 0.011). The percentage of Tregs at initial diagnosis was divided into two groups based on the median value: Tregs ≥ 2.917% and Tregs < 2.917%. There were no significant differences between the Tregs ≥ 2.917% group and the Tregs < 2.917% group in terms of the proportion of patients receiving lenalidomide as part of the treatment regimen or undergoing autologous hematopoietic stem cell transplantation. We then further analyzed their impact on survival based on these cutoff values. The results showed that the group with Tregs ≥ 2.917% appeared to have a higher PFS rate compared to the group with Tregs < 2.917% (Log‐rank *χ* ^2^ = 5.751, *p* = 0.016), refer to Figure 1e.

## 4 Discussion

The immune mechanisms are important factors leading to the development and progression of MM [13]. Basic research suggests that Tregs play a crucial role in the onset and maintenance of MM [8, 9]. Tregs can suppress the body's immune response, thereby weakening the immune attacks on tumor cells [8]. Secondly, Tregs may promote tumor growth and survival by regulating the immune response in the tumor microenvironment [8, 10]. Additionally, Tregs may also promote tumor evasion and development by suppressing the activity of other immune cells, such as CTLs and NK cells [7, 8]. Previous clinical studies showed Tregs exhibited an abnormal increase in MM [13, 14, 15]. Tregs may also be closely related to the early relapse of the disease [16]. So, it is logical to suppose that whether Tregs could be used as a clinical biomarker to predict or assess early relapse and prognosis in MM, especially in the setting of identifying FHR patients earlier except based on traditional risk stratification methods.

Early relapse is a strong predictor of inferior survival in MM and is often regarded as FHR, indicating a more aggressive disease course and poorer prognosis. Most studies have defined relapse within 18 months after initial treatment or within 12 months after stem cell transplantation (SCT) as ER18, which represents the functional high‐risk category and has a significant adverse impact on the overall survival in NDMM patients [4, 6, 17]. However, as part of FHR, ER18 is determined based on known survival outcomes, making it a retrospective survival predictor. The question remains whether there are biomarkers that can prospectively predict prognosis and serve as alternatives to ER18. Identifying such biomarkers would allow for early, individualized treatment adjustments based on prognosis.

In this study, we found that Tregs at diagnosis were important predictors of ER18 and long‐term prognosis. The presence of a lower percentage of Tregs at diagnosis was identified as a negative factor for early relapse and an independent influencing factor. By using ER18 as a marker for early relapse and FHR, we tried to explore the relationship between Tregs and ER18. Our findings revealed that patients with ER18 had a significantly reduced median OS of only 24.2 months, compared to those with later relapse. This result is consistent with previous reports indicating a nearly 2‐year median OS in the ER18 population [4, 5]. Previous studies have typically analyzed baseline clinical and biological features to identify risk factors for early relapse, including elevated LDH, extramedullary disease, and high‐risk cytogenetic abnormalities, all indicative of a high tumor burden and aggressive disease [4, 5, 17]. The above factors were also included in our study, and we found that extramedullary lesions and a lower percentage of Tregs at initial diagnosis increased the risk of early relapse by multivariate analysis. Furthermore, compared to the early relapse group, the late relapse group had a higher median percentage of Tregs at diagnosis. Our results indicate that when traditional high‐risk biological factors are incorporated, Tregs at diagnosis were demonstrated to be an independent risk factor for ER18. Therefore, Tregs at diagnosis may be used to predict some type of FHR. Previous literature has suggested a potential association between Tregs and early relapse [11]. However, to our knowledge, this is the first study to identify a correlation between Tregs at diagnosis and ER18, indicating that Tregs may serve as a potential biomarker for determining FHR.

In our study, Tregs at diagnosis are also an important factor affecting PFS. We utilized the median value as a cutoff for grouping and compared the clinical features and outcomes between higher and lower percentages of Tregs. Compared to Tregs < 2.917%, Tregs ≥ 2.917% were associated with better survival. This result is not completely consistent with the conclusions of previous studies. The study from Aref et al. [14] demonstrated that the MM subgroup of patients with higher percentages of Tregs had poor OS and PFS as compared with the MM subgroup with lower percentages of Tregs. Giannopoulos et al. [15] analyzed 66 NDMM patients; those who have higher percentages (equal or above median 6.16%) of Tregs lived significantly shorter than those with lower percentages of Tregs frequencies (below median 6.16%, *p* = 0.0134). Another study yielded similar results; Bryant et al. [18] defined MM patients with a survival time of over 10 years as long‐term survival MM (LTS‐MM) subjects. The results indicated that LTS‐MM patients showed a significant decrease in Tregs compared to MM patients (*p* < 0.005). However, regarding the changes in Tregs posttreatment, some previous studies only suggested that Tregs may increase after treatment. Gupta et al. [19] compared the Tregs between pretreatment and after 4 months of therapy; the result showed that an increase in regulatory T‐cells of CD4^+^ cells was observed in patients who responded to therapy. Muthu Raja et al. [20] had similar results in their study.

We checked and compared the results repeatedly and tried to explain the potential reasons for the discrepancies between our study and previous research mentioned before. Firstly, these are clinical studies applied in different eras and different treatments setting. Most of the previous studies were conducted before 2015, when VRd protocol as induction treatment was not popular worldwide. The first representative clinical trial on VRd protocol (Bortezomib, lenalidomide, and dexamethasone) published the first results in 2012 by EVOLUTION study [21] and then in 2017 by SWOG S0777 study [22]. The Aref et al. [14] study did not outline treatment regimens. Bryant et al. [15] mentioned in their study that treatment was predominantly with multi‐agent chemotherapy when bortezomib and lenalidomide were not widely available at that time. Giannopoulos et al. [18] did not administered bortezomib and lenalidomide in their study. In the study of Gupta et al. [19], all patients received thalidomide and dexamethasone or melphalan–prednisolone–thalidomide as therapy and had an increase in Tregs after treatment. The treatment regimen in the study by Muthu Raja et al. [20] involved only lenalidomide and dexamethasone. In our research, the treatment regimens mainly included immunomodulatory agent lenalidomide and PI bortezomib. All patients received PIs, 60.7% received lenalidomide, and no patients received thalidomide. We speculated that PI‐based treatment and the synergistic effects of PI and IMIDs may have impacts on the Tregs and change the predictive meaning of Tregs in MM patients. This may be the primary reason for the differing results.

We further discussed the effects of bortezomib and lenalidomide on Tregs in MM patients. Literature reviews indicated that there were relatively limited research on the correlation between bortezomib and Tregs. The interaction between bortezomib and Tregs is not yet clear. Ercetin et al. [23] conducted in vitro studies in mice and found that Tregs had varied effects on bortezomib, which can increase, decrease, or not change the antitumor effect of bortezomib. There is more research on the impact of lenalidomide on Tregs. In studies treated with lenalidomide‐based regimens, most studies supported that Tregs tended to increase, although some showed a decrease. Data from Raja et al. [24] indicated that the combination of lenalidomide plus dexamethasone enhanced Tregs while suppressing other immune cells. While another study yielded different results, lenalidomide appeared to alleviate tumor‐induced immune suppression by reducing systemic myeloid‐derived suppressor cells (MDSC) and Tregs [25]. Some in vitro experiments focused on B‐cell proliferative diseases confirmed that lenalidomide had various immunomodulatory effects on lymphoma B cells and CD4^+^CD25^+^ Tregs and regulated their interactions. Lenalidomide interferes with signals that induce Tregs proliferation but does not directly inhibit the proliferation of Tregs activated prior to lenalidomide treatment [26]. Therefore, the modulation of Tregs by lenalidomide may not be unidirectional and could vary with the disease status. Due to the limited number of Tregs measurements during the remission phase after treatment, we did not compare the changes in Tregs with the treatment regimen. However, based on the results from the literature, we suppose that the treatment regimens may be crucial factors in changing the Tregs and the most likely reason for the potential discrepancies between our results and those of previous studies. In the VRD treatment era, the adverse effects of a higher percentage of Tregs at diagnosis in MM patients may be overcome by the synergistic mechanisms of PIs and IMIDs. These speculations need further research for confirmation.

This paper has some limitations. The major limitation is that our study is single‐center. Secondly, although we reached a positive conclusion, our study had a limited sample size. In future research, we will continue to expand the sample size and extend the study to multiple centers. Third, our study did not include other novel drugs, including CD38 monoclonal antibodies, carfilzomib, and selinexor. Lastly, a methodological limitation concerns the phenotypic definition of Tregs. In this study, Tregs were identified as CD4^+^CD25^+^FoxP3^+^ cells, a commonly used gating strategy in flow cytometry. However, previous studies have shown that transient expression of CD25 and FoxP3 can also occur in activated conventional CD4^+^ T cells without regulatory function [27, 28]. Although such expression is typically short‐lived and may have limited impact on our results, the potential inclusion of non‐suppressive T cells cannot be completely ruled out. Incorporating additional markers such as CD127^low^ or Helios in future studies may enhance the specificity of Treg identification depending on the research objective.

In conclusion, we found that Tregs at diagnosis may be an important factor to predict early relapse and could be a biomarker to recognize FHR. In the era of PIs and IMIDs, a higher percentage of Tregs at diagnosis may indicate better PFS. We speculate that the treatment with bortezomib and lenalidomide may alter the quantity and function of Tregs in the immune microenvironment through immune modulation and its synergistic mechanisms. However, these results could not be applicable in other treatment settings simply, such as CD38 monoclonal antibodies, carfilzomib, and selinexor, and further research is needed to determine their effects. In clinical practice, monitoring Tregs could be used to track FHR and may become a part of the risk assessment for MM patients, thereby aiding in the formulation of personalized treatment plans.

## Author Contributions

**Qiaolin Zhou:** conceptualization (lead), data curation (lead), formal analysis (lead), investigation (lead), methodology (lead), project administration (lead), resources (lead), software (lead), supervision (lead), validation (lead), visualization (lead), writing – original draft (lead), writing – review and editing (lead). **Fang Xu:** conceptualization (lead), data curation (lead), formal analysis (lead), funding acquisition (lead), methodology (lead), project administration (supporting), supervision (lead), writing – review and editing (lead). **Jingjing Wen:** conceptualization (supporting), data curation (supporting), formal analysis (supporting), funding acquisition (lead), project administration (supporting), supervision (supporting). **Jing Yue:** data curation (supporting), formal analysis (supporting), software (supporting). **Ya Zhang:** investigation (supporting), resources (supporting). **Lijun Du:** formal analysis (supporting), investigation (supporting). **Kun Kou:** investigation (supporting). **Jing Su:** investigation (supporting). **Yiping Liu:** investigation (supporting). **Xiaogong Liang:** investigation (supporting).

## Ethics Statement

Ethical permission was obtained from the Ethics Committee of Mianyang Central Hospital No. S20250205‐02.

## Consent

Written informed consent to publish was obtained from study participants.

## Conflicts of Interest

The authors declare no conflicts of interest.

## Data Availability

The datasets used and/or analyzed in the current study are available from the corresponding author on reasonable request.
